# Comparison of Pipeline Embolization Device and Traditional Endovascular Therapeutic Approaches in Distal Cerebral Circulation Aneurysms Using Propensity Score Matching Analysis

**DOI:** 10.3389/fneur.2022.755122

**Published:** 2022-05-18

**Authors:** Chao Ma, Haoyu Zhu, Shikai Liang, Fei Liang, Jidian Sun, Yupeng Zhang, Chuhan Jiang

**Affiliations:** ^1^School of Clinical Medicine, Tsinghua University, Beijing, China; ^2^Department of Neurosurgery, Beijing Tsinghua Changgung Hospital, School of Clinical Medicine, Tsinghua University, Beijing, China; ^3^Interventional Neuroradiology Center, Beijing Neurosurgical Institute and Beijing Tiantan Hospital, Capital Medical University, Beijing, China; ^4^Department of Neurosurgery, Beijing Ditan Hospital, Capital Medical University, Beijing, China; ^5^Interventional Neuroradiology Center, Beijing Tiantan Hospital, Capital Medical University, Beijing, China

**Keywords:** aneurysm, complication, flow diversion, coiling, embolization

## Abstract

**Background:**

Coiling and stent-assisted coiling remain the first-line treatments for distal cerebral circulation aneurysms (DCCAs). The off-label use of the pipeline embolization device (PED) for these aneurysms has been explored recently but remains controversial.

**Objective:**

To compare traditional endovascular therapeutic approaches (coiling and stent-assisted coiling) and PED for DCCAs in a multicenter cohort of patients.

**Methods:**

A multicenter, retrospective cohort comparison study was conducted that included consecutive patients with unruptured DCCAs treated with either traditional endovascular therapeutic approaches or PED placement at three centers between 2016 and 2020. Propensity score matching analysis was applied to adjust for baseline risk factors between the PED and TET groups. Matching was based on age, sex, aneurysm size, location, morphology, adjunctive coiling, treatment history, and preoperative mRS score.

**Results:**

In total, 209 patients with DCCAs treated with PED or traditional endovascular therapeutic approaches were identified. Thirty-seven patients underwent PED treatment, and 172 patients underwent traditional endovascular therapeutic approaches. After propensity score matching, 37 aneurysm pairs were matched, and the baseline characteristics of the patients were balanced between the groups. The complete occlusion rate between PED and traditional endovascular therapeutic approach in both matched cohorts (91.7 vs. 92.3%, *p* > 0.78) was similar. The rate of periprocedural treatment-related complications in both the PED and traditional endovascular therapeutic groups was 13.5%. Univariate analysis identified average parent vessel diameter as the only predictor of complete occlusion (*p* = 0.038).

**Conclusions:**

PED is a viable option for treating DCCAs by providing occlusion and complication rates similar to those of traditional endovascular therapeutic approaches. A rigid patient selection procedure and proper planning should be undertaken to reduce treatment-related complications.

## Introduction

The pipeline embolization device (PED; Covidien, Irvine, California) is a flow-diverting stent approved for treating large or giant wide-neck proximal carotid aneurysms (1). Recently, the off-label use of PED has been extended to almost all types of cerebral aneurysms, including distal cerebral circulation aneurysms (DCCAs) located at or beyond the M1 middle cerebral artery (MCA), P1 posterior cerebral artery, and A1 anterior cerebral artery (ACA) (2). Coiling and stent-assisted coiling as traditional endovascular therapeutic approaches remain the first-line treatment for DCCAs, wherein aneurysms at these locations remain a challenge for both microsurgical and traditional endovascular therapeutic approaches (3, 4). The promising performance of PED demonstrated earlier for anatomically complex abnormalities offers a new treatment option for refractory lesions (5). The luminal reconstruction ability and avoidance of PED in jailing a microcatheter to coil the aneurysmal sac further justifies its use (6). However, PED application in these settings has particular concerns, such as the narrow parent artery diameter and the mismatch in the distal–proximal artery diameters complicating the placement of PED, which may hamper the flow diversion effect of the stent.

Although several studies have compared the safety and efficacy of the PED and traditional endovascular therapeutic approaches, this study is the first to compare PED and traditional endovascular therapeutic approaches in matched groups of patients with DCCAs (7).

## Materials and Methods

### Patient Selection

The studies involving human participants were reviewed and approved by the ethical committee of Beijing Tiantan Hospital. Written informed consent to participate in this study was provided by the participants' legal guardian/next of kin. Consecutive series of patients with unruptured DCCAs who underwent PED or traditional endovascular therapeutic approaches at three Chinese centers between March 2016 and November 2020 were included in this study. The indications for flow-diverting endovascular therapy or traditional endovascular therapeutic approaches in each case were based on medical comorbidities, complex geometrical morphology, and patient preferences. Data regarding the patients' general information, presentation, aneurysm morphology, procedural details, treatment outcomes, postoperative complications, and aneurysm occlusion at follow-up imaging were collected. Based on the outcome, every patient treated with PED was matched in a 1:1 fashion with a patient treated with traditional endovascular therapeutic approaches.

### Procedural Details

All patients were premedicated with dual antiplatelet drugs that consisted of a daily dose of 100 mg aspirin and 75 mg clopidogrel, both administered at least 7 days before the procedure. We used thromboelastography to discriminate hyporesponders to clopidogrel. The subjects who displayed an inhibition rate under 30% were deemed hyporesponsive to clopidogrel. These patients were administered a booster dose of 300 mg clopidogrel. Aspirin was continued for 12 months, and clopidogrel was discontinued 6 months after the procedure if no other coronary or cerebral comorbidities necessitated the use of antiplatelet drugs.

### Clinical and Imaging Follow-Up

Modified Rankin scores (mRS) were evaluated before treatment, on discharge, and at the last follow-up. Patient and aneurysm characteristics, procedural details, and treatment-related complications were recorded. All patients were followed up with digital subtraction angiography or computed tomography angiography, and aneurysm occlusion was graded using the 3-point modified Raymond scale.

### Statistical Analyses

Continuous variables were expressed as the mean ± standard deviation and compared using the *t*-test or Mann–Whitney *U*-test. Categorical variables were compared using the χ ^2^ test or Fisher's exact test. Statistical significance was set at *p* < 0.05. Additionally, propensity score matching (PSM) was used to balance the patients' backgrounds between the PED and traditional endovascular therapeutic groups. PSM was based on age, sex, aneurysm size, location, morphology, adjunctive coiling, previous treatment history, and preoperative mRS. We conducted a one-to-one matched analysis without replacement based on the estimated propensity score. One-to-one matched analysis used the nearest-neighbor method without replacement with the closest estimated propensity score. According to PED use and traditional endovascular therapeutic approaches followed, baseline characteristics, and operative outcomes were compared in both propensity score-matched and unmatched cohorts, respectively. Univariate analysis was used to test covariates predictive of the following dependent variables: periprocedural treatment-related complications and complete occlusion. Predictive factors found in the univariate analysis (*p* < 0.05) were entered into a multivariate conditional logistic regression analysis. Statistical analyses were performed using R 3.6.1 (Vienna, Austria; http://www.R-project.org/).

## Results

### Baseline Characteristics

The baseline characteristics and operative data for patients undergoing PED or traditional endovascular therapeutic approaches are summarized in Table 1. In all, 299 patients with 209 unruptured aneurysms were included in our study. The baseline sample included 37 consecutive patients in the PED group and 172 cases in the unmatched traditional endovascular therapeutic group.

**Table 1:** Baseline characteristics and operative data for patients undergoing PED or TET approaches.

	**Unmatched cohort**		***p*-value**	**Matched cohort**		***p-*value**
	**PED (*n* = 37)**	**TET (*n* = 172)**		**PED (*n* = 37)**	**TET (*n* = 37)**	
Age in years (IQR)^+^	53 (39–59)	57 (49–63)	0.004*	53 (39–59)	56 (50–61)	0.108
Male sex^+^	21 (56.7%)	69 (40.1%)	0.064	21 (56.7%)	18 (48.6%)	0.485
**Location of aneurysm** ^+^						
ACA	5 (13.5%)	40 (23.3%)	0.191	5 (13.5%)	6 (16.2%)	0.744
MCA	27 (72.9%)	106 (61.6%)	0.193	27 (72.9%)	24 (64.9%)	0.451
PCA	5 (13.5%)	26 (15.1%)	0.804	5 (13.5%)	7 (18.9%)	0.528
**Maximal AN diameter**^+^ **(mean** **± SD)**	12.3 (± 5.5)	7.6 (± 4.8)	<0.001*	12.3 (± 5.5)	9.2 (± 6.3)	0.005*
<5 mm	3 (8.1%)	59 (34.3%)	0.002*	3 (8.1%)	9 (24.3%)	0.058
5–14.9 mm	25 (67.5%)	101 (58.7%)	0.318	25 (67.5%)	22 (59.5%)	0.469
15–24.9 mm	8 (21.6%)	11 (6.4%)	0.009*	8 (21.6%)	5 (13.5%)	0.359
≥25 mm	1 (2.7%)	1 (0.6%)	0.323	1 (2.7%)	1 (2.7%)	1
**Previous treatment** ^+^	6 (16.2%)	3 (1.7%)	<0.001*	6 (16.2%)	3 (8.1%)	0.477
Endovascular	4 (66.7%)	1 (33.3%)	0.524	4 (66.7%)	1 (33.3%)	0.524
Microsurgical clipping	2 (33.3%)	2 (66.7%)	0.524	2 (33.3%)	2 (66.7%)	0.524
**Morphology** ^+^						
Non-saccular	29 (78.4%)	119 (69.2%)	0.265	29 (78.4%)	27 (73%)	0.588
Saccular	8 (21.6%)	53 (30.8%)	0.265	8 (21.6%)	10 (27%)	0.588
**Pretreatment-mRS** ^+^						
Good (mRS = 0–2)	37 (100.0%)	170 (98.8%)	1	37 (100.0%)	37 (100.0%)	1
Poor (mRS = 3–5)	0 (0%)	2 (1.2%)	1	0 (0%)	0 (0%)	1
**Average parent vessel diameter (mean** **±SD)**	2.3 (± 0.4)	2.6 (± 0.6)	0.025*	2.3 (± 0.4)	2.3 (± 0.4)	0.936
**Adjunctive coil placement** ^+^	9 (24.3%)	97 (56.4%)	<0.001*	9 (24.3%)	9 (24.3%)	1
**Multiple stent placement**	7 (18.9%)	9 (5.2%)	0.012*	7 (18.9%)	1 (2.7%)	0.061

Data are reported for the overall series and the propensity score-matched groups. PED, pipeline embolization device; TET, traditional endovascular therapeutic; IQR, interquartile range; ACA, anterior cerebral artery; MCA, middle cerebral artery; PCA, posterior cerebral artery; mRS, modified Rankin Scale; SD, standard deviation; *, significant result; ^+^, baseline factors used for propensity score matching.

In the PED group, most aneurysms (29/37, 78.4%) were non-saccular. MCA aneurysms were the most commonly treated lesions (27/37, 72.9%), 22 of them located on the M1 segment and five on the M2 segment. ACA and PCA aneurysms were the second most common type (5/37, 13.5%). Two were located in segment A1 and three in segment A2 for ACA aneurysms. As for PCA aneurysms, three were located in the P1 segment and two in the P2 segment. Six (16.2%) patients had recurrent aneurysms, of whom four were previously treated with primary coil embolization and two were treated with clip reconstruction. The mean maximal aneurysm diameter was 12.3 mm (± 5.5). The majority of aneurysms found had a maximum diameter of 5.0–14.9 mm (25/37, 67.5%); more giant aneurysms (15.0–24.5 mm) comprised the second largest group at 21.6%. The average parent vessel diameter was 2.3 mm (± 0.4).

In the unmatched traditional endovascular therapeutic group, most aneurysms (126/181, 69.6%) had non-saccular morphology and a maximum diameter of 5.0–14.9 mm (101/172, 57.8%). Smaller aneurysms (<5 mm) comprised the second largest group at 34.3%. Most of the aneurysms (106/172, 61.6%) were located in the MCA segment. Three (1.7%) patients had recurrent aneurysms, of whom one was previously treated with primary coil embolization and two were treated with clip reconstruction.

In the unmatched cohort, most of the baseline characteristic variables were significantly different. Aneurysms treated with PED occurred in younger patients [53 years (interquartile range, IQR 39–59) vs. 57 years (IQR 49–63), *p* = 0.004] and were larger in maximum diameter (12.3 ± 5.5 mm vs. 7.6 ± 4.8 mm, *p* < 0.001). No significant difference was found in non-saccular aneurysm morphology between the PED and traditional endovascular therapeutic groups (29/37, 78.4% vs. 119/172, 69.2%). No significant differences were observed for patients' sex, aneurysm location, and pretreatment-mRS.

After PSM, 37 aneurysm pairs were matched, and the baseline characteristics were well-balanced between the two groups. The average diameter of proximal and distal parent vessel showed a significant difference in the unmatched cohort (2.3 ± 0.4 mm with PED vs. 2.6 ± 0.6 mm with traditional endovascular therapeutic approaches, *p* = 0.025); however, after PSM, there was no significant difference in the matched cohort (2.3 ± 0.4 mm with PED vs. 2.3 ± 0.4 mm with traditional endovascular therapeutic approaches, *p* = 0.936). The aneurysm diameters of the PED and matched traditional endovascular therapeutic groups were still different (12.3 ± 5.5 mm vs. 9.2 ± 6.3 mm, respectively; *p* < 0.005); however, there was no statistical difference between the subgroups.

### Procedural Results and Angiographic Follow-Up

The applied procedures were successful in all patients in both the PED and traditional endovascular therapeutic groups, and the operative outcomes are summarized in Table 2. In the PED group, treatment with a single PED was performed in 30 cases (81.1%). Multiple devices were used in 7 cases (18.9%). Adjunctive coil placement was performed in 9 cases (24.3%). In the matched traditional endovascular therapeutic group, treatment with simple coiling was performed in 28 cases (75.7%). Stent-assisted coiling was performed in 9 cases (24.4%), and in 1 case (2.7%) two stents were used in one aneurysm. In the unmatched cohort, patients who underwent traditional endovascular therapeutic approaches experienced more adjunctive coiling treatment than those in the PED group (24.3% vs. 56.4%, *p* < 0.001) and had more previously treated aneurysms (16.2% vs. 1.7%, *p* = 0.001).

**Table 2:** Operative outcomes for patients undergoing PED or TET approaches.

	**Unmatched cohort**		***p-*value**	**Matched cohort**		***p*-value**
	**PED (*n* = 37)**	**TET (*n* = 172)**		**PED (*n* = 37)**	**TET (*n* = 37)**	
**Last angiographic follow-up**	36 (97.4%)	132 (76.7%)	0.004*	36 (97.4%)	26 (70.3%)	0.002*
Follow-up in months, median (IQR)	12.0 (6.0–12.0)	8.0 (6.0–15.0)	0.692	12.0 (6.0–12.0)	8.0 (6.0–28.0)	0.586
**Occlusion status in last follow-up**				-		
Completely occluded	33 (91.7%)	125 (94.7%)	0.777	33 (91.7%)	24 (92.3%)	1
Near completely occluded with neck remnant	3 (8.3%)	5 (3.8%)	0.488	3 (8.3%)	1 (3.8%)	0.853
Incompletely occluded	0 (0%)	2 (1.5%)	1	0	1 (3.8%)	0.419
**Treatment-related complications**	5 (13.5%)	20 (11.6%)	0.967	5 (13.5%)	5 (13.5%)	1
**Clinical follow-up**	-			-		
Good (mRS = 0–2)	35 (94.6%)	167 (97.1%)	0.306	35 (94.6%)	36 (97.3%)	0.607
Poor (mRS = 3–5)	2 (5.4%)	5 (2.9%)	0.793	2 (5.4%)	1 (2.7%)	1
Death (mRS = 6)	0 (0%)	0 (0%)	1	0 (0%)	0 (0%)	1

Data are reported for the overall series and the propensity score-matched groups. PED, pipeline embolization device; TET, traditional endovascular therapeutic; *, significant result; IQR, interquartile range; mRS, modified Rankin Scale.

In the unmatched cohort, the proportion of patients undergoing imaging follow-up in the PED group was significantly higher than in the traditional endovascular therapeutic group (36/37, 97.4% vs. 132/172, 76.7%, *p* = 0.004), while after PSM, there was a significant difference in the matched cohort (36/37, 97.4% vs. 26/37, 70.3%, *p* = 0.002). In both the unmatched and matched cohorts, there was no difference in median angiographic follow-up time between the PED and traditional endovascular therapeutic groups [median (IQR): 12 (6–12) vs. 8 (6–15) months, *p* = 0.692; and median (IQR): 12 (6–12) vs. 8 (6–28) months, respectively; *p* = 0.586]. In the PED group, 33 cases (91.7%) showed complete obliteration with O'Kelly–Marotta scale D, and 3 out of 33 (8.3%) showed near-complete occlusion with O'Kelly–Marotta scale C. In the matched traditional endovascular therapeutic group, 32/33 (92.3%) showed complete occlusion, 1/26 (3.8%) showed near-complete occlusion, and 3.8% of cases showed incomplete occlusion at the last follow-up. The complete occlusion status was similar between PED and traditional endovascular therapeutic groups in both the matched (*p* > 0.777) and unmatched cohorts (*p* = 1).

### Treatment-Related Complications and Clinical Follow-Up

Clinical data were available for all patients in both the matched groups. Treatment-related complication rates in the unmatched cohort were similar between the PED and traditional endovascular therapeutic groups (5/37, 13.5% vs. 20/172, 11.6%, *p* = 0.967), while after PSM, the complication rate was more similar (5/37, 13.5% vs. 5/37, 13.5%). Good clinical outcome (mRS = 0–2) rate at the latest follow-up did not differ between the cohorts.

In the PED group, 91.9% of patients (34/37) had good clinical outcomes at the last follow-up. Three patients experienced immediate post-procedural complications associated with a perforation infarction. The first patient experienced Heubner's perforator-territory stroke with an infarct in the left basal ganglia and centrum semiovale, presenting with mixed aphasia and right limb movement disorder. The symptoms slightly improved with intravenous administration of tirofiban; however, residual symptoms with mRS of 3 were observed at 6 months follow-up. The second patient, who had a right MCA M1 aneurysm, presented with aphasia, left central facial paralysis, and left limb hemiplegia. Digital subtraction angiography showed a diminished internal lenticulostriate artery, and computed tomography revealed new infarct in the right temporal island and basal ganglia 3 days after the procedure. The patient was discharged with central facial paralysis and severe hemiparesis (mRS 4). The third patient presented with aphasia and hemiparesis; symptoms improved with intravenous administration of tirofiban, and mRS was 1 at follow-up. One patient developed severe right hemiplegia due to acute in-stent stenosis, and the blood flow recovered after tirofiban treatment. One patient developed severe right hemiparesis due to acute parent artery thrombosis that completely recanalized after systemic tirofiban injection. The patient was discharged with mild right limb weakness and a mRS of 1 at the 6-month follow-up.

In the matched traditional endovascular therapeutic group, 97.4% of patients (36/37) had good clinical outcomes (mRS 0–2) at the last follow-up. Overall, five patients (13.5%) experienced immediate postprocedural treatment-related complications associated with cerebral infarction. Four patients experienced thrombus formation, resulting in aphasia and hemiparesis. One patient presented with small perforator occlusion that manifested as severe hemiplegia and aphasia and was left with severe dysfunction with an mRS of 3 at the 8-month follow-up. Complications in both matched groups are listed in Table 3.

**Table 3:** Treatment-related complications in the propensity score-matched groups.

	**PED**	**TET**	***p-*value**
Perforation	3 (8.1%)	1 (2.7%)	0.607
Thrombus formation	1 (2.7%)	4 (10.8%)	0.354
In-stent stenosis	1 (2.7%)	0	0.419
SAH	0	0	1

*PED, pipeline embolization device; TET, traditional endovascular therapeutic; SAH, subarachnoid hemorrhage*.

### Predictors of Aneurysm Occlusion Status and Complications

The following factors were tested as predictors of periprocedural treatment-related complications or complete occlusion: age, aneurysm size, aneurysm location, previous treatment, adjunctive coil placement, multiple stent placement, and average parent artery diameter. In the PED group, the univariate analysis revealed the average parent artery diameter as the only predictor of complete occlusion (odds ratio, 0.02; 95% CI, 0–0.79; *p* = 0.038). Multivariate logistic regression analysis did not reveal any significant factors.

## Discussion

In this retrospective matched-pair analysis, we found no significant differences in complete occlusion at follow-up and treatment-related complication rates between PED and traditional endovascular therapeutic approaches in the treatment of unruptured DCCAs.

### Angiographic Outcome

**Figure 1:**
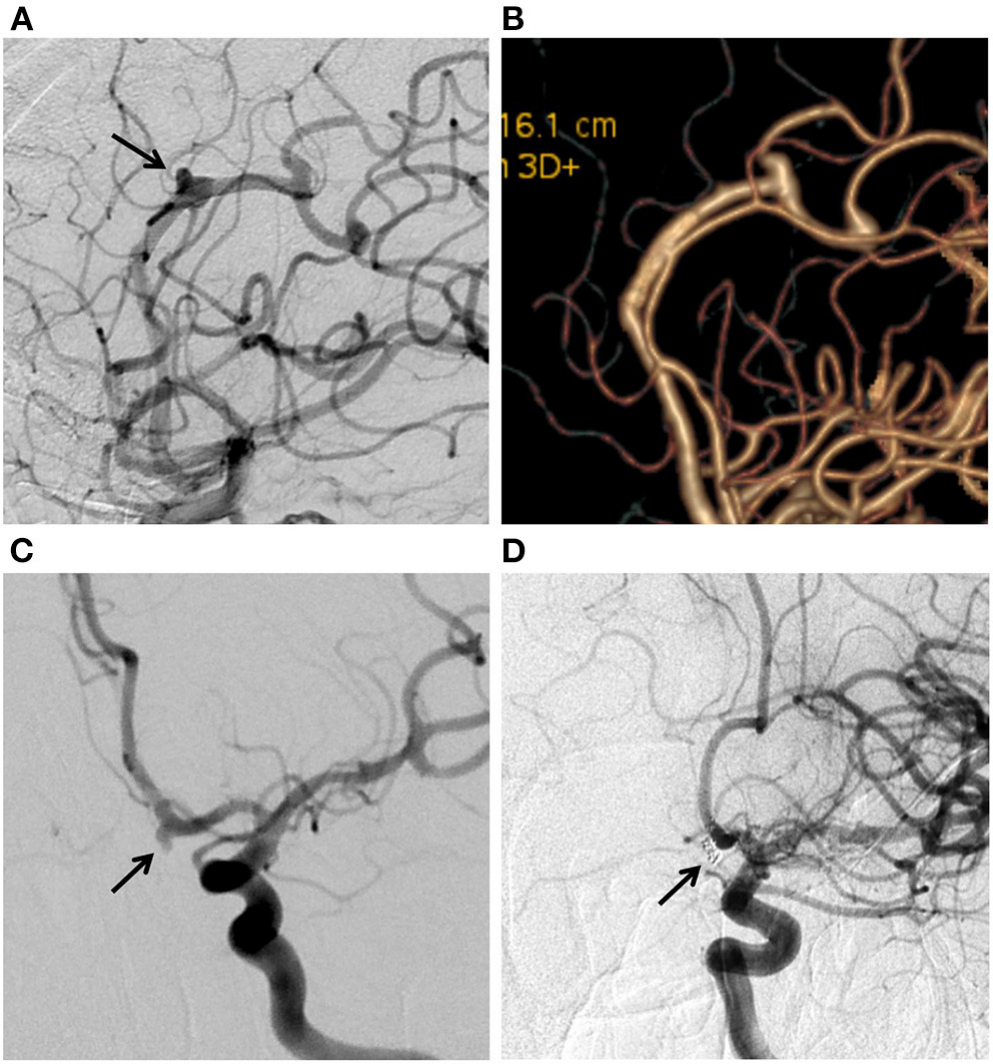
**(A)** Digital subtraction angiogram (DSA) in working position views immediately after flow diversion shows diminished filling of the aneurysms (arrow) on the ACA A2 segment. **(B)** Twelve-month DSA follow-up shows aneurysm complete occlusion. **(C)** DSA showing the ACA A1 aneurysm (arrow). **(D)** 6-month DSA follow-up shows aneurysm complete occlusion after Stent assisted coiling treatment.

Our study demonstrated a complete occlusion rate of 89.7% and a near-complete occlusion rate of 100%, which was comparable with the results of a meta-analysis of distal anterior circulation aneurysms with a median follow-up of 6 months by Cagnazzo et al. (8). This rate also appears higher than the general occlusion rate of 80% that was reported in other studies and a meta-analysis of flow diversion (FD) (9). Extensive studies have shown a high complete occlusion rate in patients with DCCAs after off-label use of PED (9). Atallah et al. (10), retrospectively reviewed 23 DCCAs treated with PED. At the last follow-up, 78.3% of patients manifested complete occlusion, and 95% had a good clinical outcome (mRS 0–2). Bender et al. (11) reviewed 67 patients with DCCAs treated with PED and reported a complete occlusion rate of 88% at 6 months, and almost 94% of patients showed a good clinical outcome. Similarly, Primiani et al. (12) found 83% complete aneurysm occlusion and 95% of patients achieving good clinical outcomes after treating 65 aneurysms at or beyond the A2, M2, and P2 segments using PED. Although traditional endovascular therapeutic approaches and PED achieved similar rates of complete occlusion in this study, there were limitations to the treatment with traditional endovascular therapeutic approaches in distal vessels. Regardless of whether simple coiling or stent-assisted coiling is used, it is not easy to deploy a catheter in a stable position at such a distal location (13). In most cases, PED avoids manipulation of the aneurysmal lumen. For example, in this study, there was an ACA segment A2 aneurysm treated with PED, which avoided unstable manipulation in the aneursymal lumen (Figures 1A,B). We also had an aneurysm in the A1 segment of the ACA that was treated with stent assisted coiling due to its relatively ideal location for catheter placement (Figures 1C,D).

As for predictors of aneurysm occlusion, Cagnazzo et al. (8) demonstrated different occlusion rates depending on the artery involved. MCA location was an independent factor for incomplete occlusion. Similarly, of the three aneurysms that were not completely occluded in our series, all were located in the M1 segment. The diameter of the MCA M1 segment was relatively larger than that of the distal artery, which may explain why only a smaller average parent artery diameter was associated with a higher complete occlusion rate in our univariate analysis.

The significant rate of recurrence treated with traditional endovascular therapeutic approaches justifies the implementation of PED for the treatment of DCCAs (14). Lin et al. (15), reviewed nine recurrent aneurysms that were subsequently retreated with PED and showed 83% complete aneurysm occlusion. Our study demonstrated comparable results, wherein 83% (5/6) of recurrent aneurysms achieved satisfactory results in the PED group. Henkes et al. (16), reported that complete occlusion was achieved in only 46.9% of retreated aneurysms after the first recoiling attempt and 35.2% after the second retreatment. Tahtinen et al. (17), focusing on the role of stent-assisted coil embolization for recurrent aneurysms, found that only 59% of aneurysms achieved complete occlusion, and 16% of patients required additional endovascular treatment. In a study by Daou et al. (18) of PED for previously coiled aneurysms, in 25% of patients, coiling was attempted twice before resorting to PED placement, which was the definitive and final treatment. The complication rates observed in these studies were comparable to those observed with the recoiling of previously coiled aneurysms. Renowden et al. (19) reported a complication rate of 3% after recoiling of recurrent aneurysms. Ringer et al. (20) reported that the total risk of retreatment mortality was 1.28% per patient. These complication rates are similar and even lower with coiling than with PED; however, in cases with multiple retreatments, the complication risk of conventional endovascular techniques may heighten with the number of reinterventions required. Overall, higher recurrence rates for previously coiled aneurysms are found with recoiling than with PED treatment, which justifies PED implementation for the treatment of previously coiled aneurysms. FD *via* PED can be positively considered as a management alternative for recurrent distal cerebral aneurysms.

The risk of in-stent stenosis (acute or chronic) must be considered when using PED in DCCAs. Two patients (7.1%, 2/28) were found to have chronic asymptomatic in-stent stenosis in our study, with 50% and 100% in-stent stenosis, respectively, at the imaging follow-up. This is comparable to the 5–10% risk in the general PED population and the 4.8% chronic in-stent stenosis rate reported by Cagnazzo et al. (8), Ravindran et al. (21) reported a rate of chronic in-stent stenosis of 7.1% after reviewing 162 intracranial aneurysms, and all these patients remained asymptomatic. Selecting a proper PED size is essential to ensure adequate FD and to limit the risk of ischemic complications. This phenomenon is common in the basilar and posterior cerebral arteries because the significant change in vascular diameter from the basilar artery to the posterior cerebral artery makes it challenging to completely open the distal end of the PED. To resolve this problem, we suggest using two PEDs of different sizes to treat fusiform or dissecting aneurysms with a wide aneurysm sac neck. However, multiple stents increase the metal coverage, which mitigates FD while also increasing the risk of in-stent stenosis (22). Therefore, it is imperative to recognize the native anatomy of the distal vessel to select an appropriately sized PED.

### Treatment-Related Complications

The effectiveness of PED must be weighed against the risk. Our study showed relatively higher treatment-related complication rates of 13.5% (5/37) compared with other reports of DCCAs, with 5.4% (2/37) morbidity. Five patients presented with immediate post-procedural cerebral infarction. Most symptoms had improved at discharge or clinical follow-up, with mRS scores of 0–1. In both the matched groups, we did not observe any new permanent neurological deficits at follow-up.

Regarding potential ischemic complications for ACA A1 segment aneurysms, we should consider the perforating medial lenticulostriate vessels. We found one ACA A1 aneurysm in which the recurrent artery of Heubner was jailed, and the patient experienced permanent symptomatic perforator-territory ischemia. However, some studies have indicated that the diameter of the recurrent artery of Heubner approximates to that of the ophthalmic artery and anterior choroidal artery, vessels that can be safely jailed when PED is used in patients with distal ICA aneurysms (22). PED used in ACA aneurysms may also induce perforator occlusion, especially at the A1-A2 junction (23, 24).

Our study, which included 27 MCA aneurysms, demonstrated a perforator-territory ischemia rate of 7.4% (2/27 patients) after the coverage of lenticulostriate vessels in the M1 segments. Kathryn et al. (25) reported a similar rate of 9.6% (5/52 patients) after the coverage of MCA M1 segments by flow diverters, but none had radiographic infarcts in the lenticulostriate territory. A study also focused on the covered perforator vessels of circle of Willis aneurysms treated by a flow diverter and showed a rate of temporary ischemic complications of 17.6% (3/17 patients) (26). Branching vessels and perforators arising from aneurysms are abundant at the level of the A1 segment and the M1 segment of the ACA and MCA, respectively, thereby increasing the risk of perforator stroke when they are covered with PED (27). Regarding non-perforator areas, Primiani et al. (12) analyzed 65 patients with A2, M2, P2, and distal aneurysms treated with PED. In their study, the overall complication rate was 7.7%, which was significantly lower than that found in our study. Furthermore, only one patient (1.5%) with an M2 aneurysm showed ischemic stroke and slow filling of the side branch, which resolved after administration of a IIb/IIIa inhibitor.

Asymptomatic occlusion of covered cortical branches appears universal, yet, ischemic complications are preferably linked to lenticulostriate territory occlusions. The fact that in our study, three of these events led to patient neurological deficits highlights the importance of awareness that these complications can occur at any time during the endovascular procedure, especially in the MCA M1 and ACA A1 segments. It is important to know how to respond in every possible situation and to be prepared. The overall complication rates with PED were similar to those found in the traditional endovascular therapeutic group. However, there were some differences in the types of complications between the two groups. Perforator-territory ischemic events were more common in the PED group, whereas thrombus formation was more common in the traditional endovascular therapeutic group (Table 3).

Several studies have also reported low hemorrhagic complication rates in PED for intracranial aneurysms (28). However, delayed aneurysm rupture has been reported after treatment with PED, and this is one of the major concerns. Brinjikji et al. (29) reported that the incidence of delayed aneurysmal subarachnoid hemorrhage after PED was ~4%. Some hemodynamic studies have attempted to explore the mechanism of delayed aneurysm rupture. Hassan et al. (30) found that a slow blood flow jet still exists inside the aneurysm at the end of the procedure. Cebral et al. (31) reported that PED placement could increase intra-aneurysmal pressure. Similarly, Li et al. (32) found that the luminal flow velocity was decreased in aneurysms with delayed rupture, while the pressure was increased. These factors may be related to delayed aneurysm rupture after treatment. For some large or huge aneurysms, a combination treatment of PED placement and coil embolization of the aneurysm has been recommended to promote intraluminal thrombosis and the transition from an unstable thrombus to a stabilized, organized thrombus (33).

### Limitations

This study has several limitations, including those inherent to a retrospective observational series, such as the limited number of cases and the relatively short follow-up period. While both PED and traditional endovascular therapeutic cohorts constituted consecutive cases, data collection and analysis were performed retrospectively and, as such, were subject to incomplete datasets. Extensive studies with long-term follow-up are needed to confirm the safety and efficacy of PED in DCCAs.

## Conclusions

PED treatment is a reliable and safe alternative for the treatment of DCCAs, especially in the case of recurrent aneurysms or those that are not amenable to traditional surgical or endovascular modalities. Proper planning and stringent patient selection may lead to better clinical outcomes.

## Data Availability Statement

The raw data supporting the conclusions of this article will be made available by the authors, without undue reservation.

## Author Contributions

CM and HZ acquired most of the data, analyzed and interpreted the data, and drafted the article. SL, FL, and JS participated in the interventional procedures as assistants and helped to analyze the data. CJ and YZ participated in the interventional procedures as primary surgeons and made substantial contributions to the design of the work. All authors contributed to the article and approved the submitted version.

## Funding

This work was supported by the National Natural Science Foundation of Beijing Grant Number 7212007.

## Conflict of Interest

The authors declare that the research was conducted in the absence of any commercial or financial relationships that could be construed as a potential conflict of interest.

## Publisher's Note

All claims expressed in this article are solely those of the authors and do not necessarily represent those of their affiliated organizations, or those of the publisher, the editors and the reviewers. Any product that may be evaluated in this article, or claim that may be made by its manufacturer, is not guaranteed or endorsed by the publisher.

## Abbreviations

ACA: anterior cerebral artery
MCA: middle cerebral artery
PED: pipeline embolization device
DCCAs: distal cerebral circulation aneurysms
IQR: interquartile range
mRS: modified Rankin Scale
FD: flow diversion
TET: traditional endovascular therapeutic.
